# Publisher Correction: Evolution of enhanced innate immune evasion by SARS-CoV-2

**DOI:** 10.1038/s41586-022-04653-w

**Published:** 2022-03-24

**Authors:** Lucy G. Thorne, Mehdi Bouhaddou, Ann-Kathrin Reuschl, Lorena Zuliani-Alvarez, Ben Polacco, Adrian Pelin, Jyoti Batra, Matthew V. X. Whelan, Myra Hosmillo, Andrea Fossati, Roberta Ragazzini, Irwin Jungreis, Manisha Ummadi, Ajda Rojc, Jane Turner, Marie L. Bischof, Kirsten Obernier, Hannes Braberg, Margaret Soucheray, Alicia Richards, Kuei-Ho Chen, Bhavya Harjai, Danish Memon, Joseph Hiatt, Romel Rosales, Briana L. McGovern, Aminu Jahun, Jacqueline M. Fabius, Kris White, Ian G. Goodfellow, Yasu Takeuchi, Paola Bonfanti, Kevan Shokat, Natalia Jura, Klim Verba, Mahdad Noursadeghi, Pedro Beltrao, Manolis Kellis, Danielle L. Swaney, Adolfo García-Sastre, Clare Jolly, Greg J. Towers, Nevan J. Krogan

**Affiliations:** 1 Present Address: Division of Infection and Immunity, University College London, London, UK; 2 QBI Coronavirus Research Group (QCRG), University of California San Francisco, San Francisco, CA USA; 3 Quantitative Biosciences Institute (QBI), University of California San Francisco, San Francisco, CA USA; 4 J. David Gladstone Institutes, San Francisco, CA USA; 5 Department of Cellular and Molecular Pharmacology, University of California San Francisco, San Francisco, CA USA; 6 Division of Virology, Department of Pathology, Addenbrooke’s Hospital, University of Cambridge, Cambridge, UK; 7 Epithelial Stem Cell Biology and Regenerative Medicine Laboratory, The Francis Crick Institute, London, UK; 8 MIT Computer Science and Artificial Intelligence Laboratory, MIT, Cambridge, MA USA; 9 Broad Institute of MIT and Harvard, Cambridge, MA USA; 10 European Molecular Biology Laboratory (EMBL), European Bioinformatics Institute, Wellcome Genome Campus, Hinxton, UK; 11 Department of Microbiology, Icahn School of Medicine at Mount Sinai, New York, NY USA; 12 Global Health and Emerging Pathogens Institute, Icahn School of Medicine at Mount Sinai, New York, NY USA; 13 Howard Hughes Medical Institute, San Francisco, CA USA; 14 Division of Advanced Therapies, National Institute for Biological Standards and Control, South Mimms, UK; 15 Present Address: Cardiovascular Research Institute, University of California San Francisco, San Francisco, CA USA; 16 The Tisch Cancer Institute, Icahn School of Medicine at Mount Sinai, New York, NY USA; 17 Department of Medicine, Division of Infectious Diseases, Icahn School of Medicine at Mount Sinai, New York, NY USA; 18 Department of Pathology, Molecular and Cell-Based Medicine, Icahn School of Medicine at Mount Sinai, New York, NY USA

**Keywords:** SARS-CoV-2, Systems biology

Correction to: *Nature* 10.1038/s41586-021-04352-y Published online 23 December 2021

In the version of this article initially published, there were labeling errors in Figs. 2 and 3. In Fig. 2c, the labels “10 h” and “24 h” were omitted from atop the left and right-hand lanes of the heatmap, respectively. In Fig. 3i, the *y*-axis label, now reading “Alpha sgRNA reads”, initially appeared as “Alpha RNA reads”. The errors have been corrected in the HTML and PDF versions of the article

